# 

**Published:** 1872-06

**Authors:** 


### INDEX OF SUBJECTS.

### ORIGINAL COMMUNICATIONS.

Variola........................................................................321
Chronic Cystitis—Perineal Cystotomy............................................328
Nostrums.......................................................................333
Quine in Spasms with Hooping Cough.............................................336
Enlarged Prostate..............................................................337
Correspondence.................................................................340
Explanation of Two Well Known Symptoms of Phthisis.............................341
Operations upon Hemorrhoids....................................................343
On the Treatment of Some Forms of Menorrhagia..................................345
On Dysmenorrhœa................................................................350

### EXTRACTS AND GLEANINGS.

Recipes, 354; Chronic Diarrhœa, Sequel of Typhoid Fever, 355; Styptic Cotton, 356; Chronic Catarrh of the Uterus, 357; Arsenic in Dyspepsia—Arsenic in Irritable Stomach, 359; Acne Pustulosa, 360; Hypophosphites of Iron, Quinia, and Strychnia, in Cases of General Debility and Nervous Exhaustion—Celery as a Nervine—Seton Treatment of Serous Tumors, 361; Beef Tea—Ice in Acute Rheumatism—Canula for Tracheotomy, 362; Tinctures Podophilum, Dandelion, and Phytolacca—To Administer Pills—Treatment of Ozæma, 363; Alcohol—Charcoal in Gastric and Enteric Disorders, 364; Splenitis and Chronic Ague—Professor Sayre's Vertebrated Probe and Catheter, 365; Sulpho-Carbolate of Soda, 366; Indication for the Employment of the Catheter in Old Persons, 367; Vomiting of Pregnancy Treated by Electricity—Chloral in Gout—Chloride of Ammonia in Hepatitis and Hepatitic Abscess, 368

### EDITORIALS, REVIEWS, ETC.

Our "Chesterfield" Again.......................................................369
Bibliographical................................................................377
The American Medical Association...............................................380

